# Tubular Adenoma of the Breast: A Rare Presentation and Review of the Literature

**DOI:** 10.4021/jocmr746w

**Published:** 2012-01-17

**Authors:** Nikolaos S. Salemis, Georgios Gemenetzis, Gregorios Karagkiouzis, Charalambos Seretis, Konstantinos Sapounas, Vlasios Tsantilas, Dimitrios Sambaziotis, Emmanuel Lagoudianakis

**Affiliations:** a Breast Surgery Unit, Army General Hospital, Athens, Greece; b The 2nd Department of Surgery, Army General Hospital, Athens, Greece; c Department of Pathology, Army General Hospital, Athens, Greece

## Abstract

Tubular adenomas, also known as pure adenomas, are rare epithelial tumors of the breast. Only a few cases have been reported in the literature, especially in young women of reproductive age. Postmenopausal women are very rarely affected. We describe here a very rare case of tubular breast adenoma in a postmenopausal woman who presented with a gradually enlarging breast lump. Clinical examination and imaging studies revealed a non-tender well circumscribed left breast tumor suggestive of a fibroadenoma. Due to the history of progressive enlargement of the breast lump, a surgical excision was performed. Histological findings were suggestive of a tubular breast adenoma. We conclude that although tubular breast adenoma is rare, it should always be considered in the differential diagnosis in postmenopausal patients presenting with a gradually enlarging breast mass. Preoperative diagnosis is difficult because tubular adenoma is indistinguishable from a fibroadenoma on physical examination and breast imaging. Surgical excision is necessary to establish a definitive diagnosis. Clinical presentation and management of our patient are discussed along with a review of the literature.

**Keywords:**

Tubular adenoma; Breast; Breast mass.

## Introduction

Tubular adenoma is a rare benign epithelial tumor of the breast accounting for 0.13 - 1.7% of benign breast lesions [1]. It was first described as a distinctive entity in 1968 by Persaud et al. [2]. The first case of tubular adenoma of the breast studied by aspiration cytology and light and electron microscopy was reported by Moross et al in 1983 [3]. Few cases have been reported in the literature especially in young women of reproductive age [4]. The clinical and imaging features of tubular breast adenomas are similar to those of fibroadenomas [1], thus making preoperative diagnosis very difficult. In most cases surgical excision is required to establish a definitive diagnosis. We herein describe a very rare case of a gradually enlarging breast tubular adenoma in a 50-year-old postmenopausal woman. Diagnostic evaluation and management are discussed along with a review of the literature.

## Case Report

A 50-year-old woman presented to our Breast Unit complaining of a gradually enlarging palpable mass in the middle outer portion of her left breast, that she first noticed 3 months ago. She had then undergone an ultrasonography at another institution which showed a mass measuring 1.1 × 1 cm with imaging features suggestive of a fibroadenoma. Her past medical history was significant for rheumatoid arthritis and asthma, whereas she had undergone an appendectomy and cesarean section twice at 25 and 28 years of age. She had no family history of breast or ovarian cancer whereas she had gone through the menopause at the age of 45 without taking any hormone replacement therapy.

**Figure 1:**
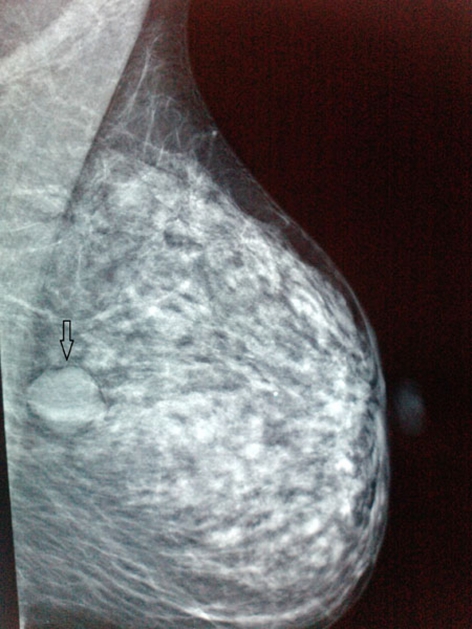
Left mediolateral oblique mammogram shows a multilobulated, well-circumscribed mass without calcifications, suggestive of a fibroadenoma.

**Figure 2:**
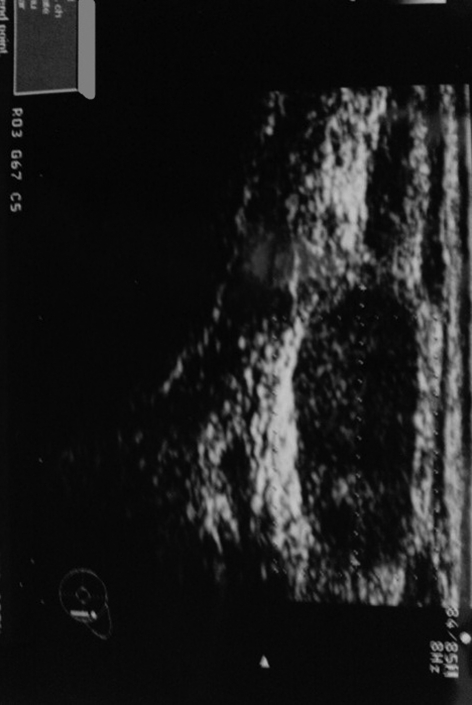
Ultrasonography shows a slightly lobulated hogogenous hypoechoic mass with slight posterior acoustic enhancement, without calcifications.

On physical examination, a non-tender, mobile well-circumscribed mass measuring approximately 2.5 cm × 2 cm was palpated at the middle outer portion of the left breast. There were no skin alterations or nipple discharge whereas there were no palpable axillary or supraclavicular lymph nodes. Mammogram showed a multilobulated well-circumscribed mass without any calcifications (Fig. 1) whereas a breast ultrasound showed an oval hypoechoic mass measuring 2.2 × 2 cm with mild degree of posterior acoustic enhancement (Fig.2). The aforementioned imaging characteristics were suggestive of a fibroadenoma.

**Figure 3:**
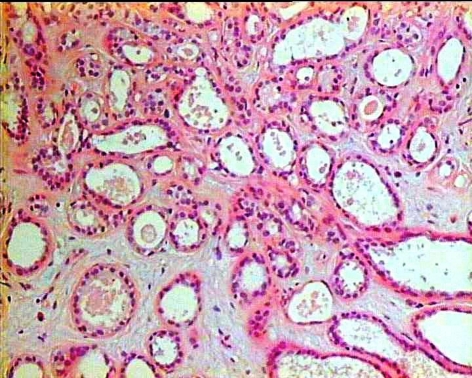
Photomicrograph showing closely approximated round or oval glandular structures. The glandular proliferation has a pattern that resembles tubular adenosis. There is a single layer of epithelium supported by a layer of myoepithelial cells. A small amount of secretion is present in the glandular lumens H&E stain, (original magnification × 100).

Since the mass had over doubled in size over the previous 3 months, a wide local excision was performed. At surgery a well-circumscribed oval demarcated mass measuring 2.5 cm was easily resected along with a small cuff of surrounding tissues. Macroscopically, the tumor measuring 2.2 × 2.1 × 2 cm presented as a solid white elastic nodule with a smooth surface resembling a fibroadenoma. Histological examination of the mass revealed tubular breast adenoma. Closely approximated round and oval glandular structures composed of a single layer of epithelium and supported by a layer of myoepithelial cells were noticed. Little presence of stroma with mild fibrosis and myxoid degeneration was apparent whereas a small amount of secretion was present in the glandular lumens (Fig. 3). The patient had an uncomplicated postoperative course. She is doing well, without any evidence of recurrence 18 months after surgery.

## Discussion

Breast adenomas are pure epithelial neoplasms. According to the classification proposed by Hertel et al. [5] breast adenomas are subdivided into true ademomas, nipple adenomas and fibroadenomas. Tubular breast adenomas or pure adenomas are rare epithelial tumors that belong to the class of adenomas [5,6]. They are considered variants of pericanalicular fibroadenomas with an exceptionally prominent or florid adenosis-like epithelial proliferation [7].

Tubular breast adenomas most often affect young women or reproductive age [1], and have not been associated with oral contraceptive treatment or pregnancy [5]. In 90% of the cases these tumors are found in patients younger than 40 years old [6], whereas the elderly women are very rarely affected [4,8]. Nagata et al. [9] reported that out of 32 cases of tubular breast adenoma reported in the Japanese literature only 2 occurred in women older than 65 years. Extremely rare cases have been reported in juvenile women [10] and in pregnant women with rapid tumor enlargement [9].

Tubular adenomas usually represent painless freely movable well-defined breast masses without associated skin or nipple alterations [5,8] and clinically resemble fibroadenomas [7,11].

Their size varies from 1 to over 7.5 cm [11] and they may be present for 2 - 12 months before a diagnosis is made [12]. Grossly, tubular adenoma is well-circumscribed with solid homogenous to finely nodular tan yellow cut surface and firm consistency [1], and tends to be softer than fibroadenoma [7]. Tavassoli et al. [11] required for a nodule to qualify as a tubular adenoma, to be at least 1cm in size or encapsulated if smaller.

Histologically, the tumor is characterized by the presence of tightly packed homogenous tubular and acinar epithelial components with sparse intervening stroma on the contrary to fibroadenoma which contains a large amount of stroma [6]. Tubular lumens are small and empty but sometimes may contain eosinophilic proteinaceous material [1], as seen in our case. Focal or extensive infarction has been reported in 2.4% of the cases [11] but hemorrhage or necrosis has not been observed [5]. Maiorano and Albrizio [13] studied 10 cases of tubular adenomas and 6 cases of fibroadenomas in order to investigate possible relationships between these 2 tumors. They found that the morpholological characteristics of tubular adenoma closely resemble in some areas of the tumors those of fibroadenoma and they suggested that the 2 tumors may are histogenetically related with predominant stromal component in fibroadenomas and exuberant ductular component in tubular adenomas [13].

Very rare cases of in situ or invasive cancers have been reported to develop in tubular breast adenomas [1]. Domoto et al. [14] reported a case of synchronous occurrence of a tubular adenoma with a ductal invasive breast carcinoma. Histology in that case showed that a boundary was clearly defined between the tubular adenoma and the ductal carcinoma and between tubular adenoma and adjacent breast tissue. The authors stated that although that case might be a collision between the 2 tumors, a malignant transformation of a tubular ademoma could not be ruled out [14]. Komaki et al. [15] reported a case where histology revealed the presence of 2 separate patterns that of tubular adenoma and fibroadenoma in an excised breast mass. These 2 patterns were distinct and there was no transitional zone suggesting that the 2 tumors are closely related to each other.

Histologically, the differential diagnosis of tubular adenomas includes fibroadenoma, nipple adenoma, sclerosing adenosis, eccrine spiradenoma and tubular carcinoma [5].

There are only a few reports on fine needle aspiration cytology (FNA) of tubular adenomas. The main findings on FNA cytology include cells arranged in small, three- dimensional balls or clusters and tubular structures with or without closely approximated acini [12]. Kumar et al. [16] compared FNA smears from 6 histologically documented cases of tubular adenoma with 10 histologically confirmed cases of fibroadenoma and reported that an initial cytological diagnosis of tubular adenoma was made only in one case. Differential diagnosis based on cytological features of tubular adenomas includes adenosis tumor and tubular carcinomas when tubular fragments are present [12].

A precise diagnosis is even more difficult in the presence of associated features such as mucinous secretion [17]. In addition, the presence of degeneration or infarction may be associated with atypia that can mimic malignancy in FNA smears [18,19]. Awareness of the tubular adenoma and its characteristics is therefore needed in order to prevent unnecessary aggressive treatment [19,20].

Preoperative diagnosis of tubular adenoma is very difficult because in most cases the imaging features are non-specific and are similar to those of fibroadenoma. In rare cases however the radiologic findings may be suggestive of a malignant lesion [4,17]. Soo et al. [21] studied the imaging features of 17 patients and found that in younger women tubular adenomas resemble non calcified fibroadenomas on both mammography and ultrasonography but in older women microcalcifications may be present and the tumor may resemble a malignancy thus making biopsy necessary. Tubular adenoma is a completely benign tumor and has not been associated with an increased risk of breast cancer development [11]. In many cases however, surgical excision is necessary to obtain a definitive diagnosis [4].

### Conclusions

Tubular adenomas of the breast are rare benign epithelial lesions that are most commonly found in young women of reproductive age. Preoperative diagnosis is difficult because in most cases the clinical findings and imaging features resemble fibroadenomas. The development of a tubular adenoma in postmenopausal women, as a progressively enlarging breast mass, as seen in our case, is a very rare occurrence. Surgical excision is necessary to obtain a definitive diagnosis.
